# A Study on the Microstructure and Properties of CoCr Alloy Deposited Via Arc Deposition on a Single-Crystal Alloy

**DOI:** 10.3390/ma18173994

**Published:** 2025-08-26

**Authors:** Shuai Huang, Tianyuan Wang, Jian Miao, Cheng Wang, Wei Liu, Guohui Zhang, Bingqing Chen, Biao Zhou

**Affiliations:** 3D Printing Research and Engineering Technology Center, Beijing Institute of Aeronautical Materials, Beijing 100095, China

**Keywords:** welding wire, deposition layer, properties, microhardness

## Abstract

Three types of CoCr alloy welding wires were deposited on the surface of a single-crystal alloy using gas tungsten arc-welding (GTAW) technology to enhance its wear resistance. Comparative studies were conducted on the microstructure, microhardness, friction and wear properties, and tensile properties of the deposited alloy layers. The results showed that the deposited CoCr alloy layers formed good metallurgical bonding with the substrate, and the microstructure mainly consisted of planar crystals, coarse columnar dendrites, and fine, dense equiaxed dendrites. The microhardness of joints formed by depositing CoCr alloy welding wires increased with increasing distance from the interface, exhibiting a distribution pattern where the center of the deposition layer had the highest hardness, followed by the interface, while the base material had the lowest hardness. The highest hardness of the deposited layers of the S1 alloy was 119.5 HRC, with significant fluctuations in the deposition layer area. The wear resistance was significantly improved after depositing, and decreased significantly with increasing service temperature. The tensile strengths of the three welding wires were similar. The joint strength gradually decreased as the test temperature increased, with the S12 alloy joint exhibiting superior performance at high temperatures.

## 1. Introduction

Single-crystal alloys have excellent compressive strength and thermal strain capacity and are mainly used to produce high-speed rotating components in new aircraft power plants [1,2]. Due to the high temperature and pressure friction between the meshing serrated crowns during operation, wear between meshing surfaces can easily occur, resulting in a decrease in the working efficiency. Therefore, it is necessary to prepare a wear-resistant layer between the meshing serrated crowns to improve their wear resistance, reduce wear, and extend the service life [3,4,5,6].

Co-based alloys are widely used in surface-modified coating materials due to their excellent mechanical properties. The preparation methods of Co-based alloy coatings mainly include plasma spraying [7], supersonic flame spraying [8], laser cladding [9,10,11,12], electrochemical deposition [13], arc welding [14], and other methods. Arc welding [15] uses high-density arcs in a closed circuit to melt the substrate and welding wires, and metallurgical reactions occur between the substrate and wires to form a strong bonding interface. At the same time, the carbon in the welding wire or coating reacts with oxygen in the air to produce carbon monoxide gas, which stirs the melt pool and helps to homogenize the coating’s composition and structure. At the interface between the coating and the substrate, the rapid cooling effect of the substrate generates a large temperature gradient, which helps to form columnar crystals perpendicular to the interface. At the far interface of the coating, the grains inside the coating exhibit equiaxed crystalline states due to the small temperature gradient. In the middle of the coating, the coating structure includes columnar crystals and equiaxed crystals [16]. A coating with a thickness of 1–30 mm, which has different mechanical properties from the substrate and enhances its wear, corrosion, and oxidation resistance, can be deposited on the substrate surface using this technology [17,18].

Arc welding is an efficient, reliable, and versatile welding technology that is suitable for various industrial applications and can significantly improve production efficiency and product quality. Zhu et al. [19] applied this technology to weld Stellite 6 alloy onto heavy-duty engine valves, increasing the surface hardness of the valves to 390HV, thus meeting the requirements of heavy-duty engine valves. In addition, arc-welding technology is used to repair failed high-temperature alloy components and extend their service life. Chen et al. [20] compared the characteristics of laser-cladding technology and arc-welding technology and found that arc-welding technology has the features of high efficiency, low cost, and easy operation. Egerland et al. [21] explored the application and effectiveness of gas tungsten arc-welding (GTAW) technology and found that dual-anode GTAW can significantly improve the welding deposition rate and production efficiency. Lolla et al. [22] found that cracks appeared at the interface between a Stellite 21 coating and P91 substrate steel, and the crack microstructure was layered with a hardness of 600 HV_0.3_, far exceeding the hardness of the substrate and coating. Wang et al. [23] revealed the mechanism of Stellite 21 coating failure on COSTE steel’s surface before and after service. The interface structure between the as welded Stellite 21 alloy coating and COSTE steel is mainly composed of α-Co solid solution. After service, the interface structure transforms into a two-phase structure of α-FeCo (Cr, Mo) and σ-CrCo (Fe, Mo), and the hardness of the interface layer after service is higher than that of the as welded interface layer.

The optimization of welding process parameters is commonly conducted to improve coating performance. Such optimization can enhance the bonding strength and coating performance between the Stellite coating and the substrate. However, the improvement effect on the alloy coating performance is limited [24,25]. Bharath et al. [26] deposited Stellite F alloy coatings using arc-welding technology and studied the effects of welding parameters on the coatings. A high welding current may increase the dilution rate of the coating and reduce the hardness of the alloy coating. An appropriate preheating temperature can reduce cracks during the coating deposition process. Ciubotariu et al. [27] deposited a Stellite 6 coating on a martensitic stainless-steel substrate using HVOF thermal-spraying technology and optimized the microstructure and properties of the coating by combining laser-remelting technology. HVOF technology heats materials to a semi-molten state through a high-speed flame flow, forming coatings with high density and a high bonding strength, making it especially suitable for spraying hard materials such as carbides. However, the equipment and maintenance costs are high, the process requires a large amount of fuel, and the noise is high, with strict requirements for the operating environment [28].

Doping treatment can improve the wear resistance and erosion resistance of Stellite alloy coatings, but this increases the complexity of the process and has uncertain effects on the oxidation resistance, creep resistance, and other properties of the alloy coatings. Cui et al. [29] used a ball mill to mix CeO_2_ powder and Stellite 6 powder, and used laser cladding technology to melt the mixed powder onto the surface of 42CrMo steel. CeO_2_ powder refined the alloy coating grains, reduced the residual stress of the alloy coating, and effectively reduced the crack sensitivity of the alloy coating. Among them, when 3% CeO_2_ powder was added, the wear rate of the alloy coating decreased to 50% of the original alloy coating.

Using gas tungsten arc-welding (GTAW) technology to prepare wear-resistant layers on alloy surfaces can effectively improve the surface wear resistance. However, the uncertainty of process parameters and changes in material composition lead to changes in the microstructure and properties of the deposited layer. The mechanical properties of the wear-resistant layer can be further improved using the method of compositional changes, but there is limited research on the preparation of a CoCr wear-resistant layer on the surface of a single-crystal alloy. The influence of different microstructures on the performance of the deposited CoCr alloy layers requires further analysis. This work researched the preparation of a deposited CoCr layer on the alloy surface using GTAW. The influence of welding wires with different compositions on the deposited layer was compared. The microstructural characteristics of the deposited layer were observed, and its hardness distribution was analyzed. The hardness and friction wear properties were tested, providing a reference for the laser deposition of a CoCr layer on a single-crystal alloy.

## 2. Materials and Methods

The matrix material was DD5 single-crystal alloy. The welding wire was Stellite alloy, which is a widely used cobalt alloy with high strength, hardness, corrosion resistance, erosion resistance, and wear resistance. Three types of Stellite alloy were selected as welding wires for the experiment, with a diameter of 1.6 mm. The specific composition and melting point are shown in Table 1. Tungsten is the metal with the highest melting point, forming tungsten carbide with high hardness, a high melting point, and high brittleness. The formed carbides were stable and could form various carbides due to the strong interaction between tungsten and carbon, improving the hardness of the matrix [30].

Before the welding test, the surface was polished to a bright finish and cleaned with anhydrous ethanol. When welding, the qualified process parameters were evaluated according to the ASMEBPVC.IX standard [31] for GTAW. Tungsten electrode gas-shielded arc welding was carried out in two layers and multiple passes to ensure that the thickness of the deposited alloy layer was greater than 3.0 mm. After welding, the insulation was slowly cooled to room temperature, and the surface of the deposited layer was visually inspected to ensure there were no visible defects. After passing, the deposited layer was turned and ground, and then samples were taken and prepared horizontally and vertically in the scanning direction, ensuring the effective thickness of the deposited layer.

The microstructure and composition distribution of the cross-section of the deposited layer were observed using a scanning electron microscope (Sigma, ZEISS, Oberkochen, Germany). Phase testing was performed using an X-ray diffracatometer (D8 Discover, Bruker, Billerica, MA, USA) on the alloy layer’s surface with a tube voltage of 30 kV, a tube current of 20 mA, a scanning angle of 20–90°, and a continuous scanning rate of 0.03°/s. A digital microhardness tester was used to perform hardness testing, with a load weight of 300 g and a holding time of 10 s. During the testing process, based on the corrosion traces as much as possible, dot tests were conducted every 100 μm along a straight line from the top of the deposited layer to the substrate to ensure that the hardness of the deposited layer top, middle, bottom, bonding area, and substrate was accurately tested. The average value was taken from five tests per group of data. During the friction and wear test, the surface of the sample to be tested was subjected to rough grinding, fine grinding, and polishing treatment using metallographic sampling methods to reduce the influence of the surface condition on performance. The friction and wear test utilized an MFT-5000 multifunctional reciprocating friction and wear testing machine (Retc-Instruments, Yverdon-les-Bains, Switzerland) at room temperature with no medium friction in the atmosphere, with a loading force of 30 N, a frequency of 2 Hz, a stroke of 5 mm, a time of 30 min, and real-time detection of the wear depth and wear volume during the friction and wear process.

## 3. Results and Discussion

### 3.1. Microstructural Characteristics of Three Types of Welding Wires

Stellite alloy is a popular type of Co-based high-temperature alloy, which contains a cobalt matrix and a high content of Cr (20–30 wt. %), a moderate content of W or Mo (4–18 wt. %), and a small amount of C (0.25–3 wt. %). It has excellent mechanical properties, especially under high-temperature conditions [32]. The main difference between S6 and S12 lies in their different carbon contents. Alloys with different carbon contents can be used in different situations, such as low-carbon alloys that can be used for cavitation, sliding wear, and medium–low-degree anti-wear applications. High-carbon alloys can be used in higher-degree wear-resistant applications. Their excellent wear resistance is mainly due to the good wear resistance of the carbides embedded in the cobalt matrix. Co-based high-temperature alloys are carbide-reinforced, and the carbide content formed by different carbon contents varies, which makes the material have different properties. S1 has a maximum carbon content of 1.95 wt. %, making it the most wear-resistant type of Stellite alloy, but it is the most prone to cracking. The carbon content of S12 is 1.5 wt. %, and its good wear resistance is increasingly valued.

**Figure 1:**
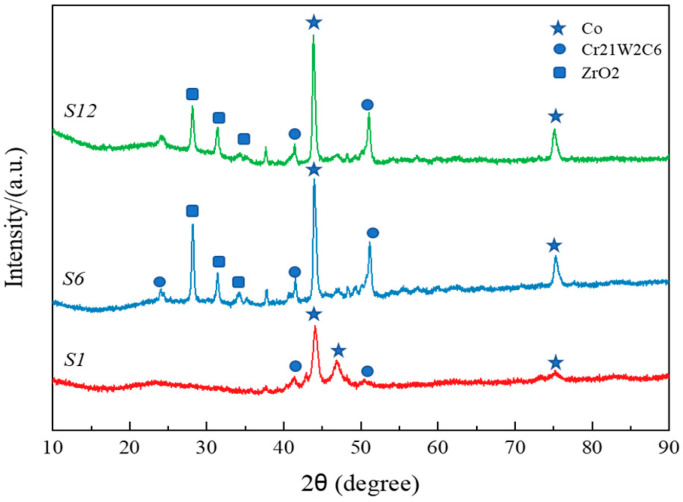
XRD patterns of three types of welding wire materials.

Figure 1 displays the XRD pattern of the Stellite alloy layer. The XRD analysis results show that S1 mainly contained Co (a cubic crystal system), Cr21W2C6 (a cubic crystal system), and Co (a hexagonal crystal system). The S6 sample mainly contained Co (a cubic crystal system), Cr21W2C6 (a cubic crystal system), ZrO_2_ (a monoclinic crystal system), and Co (a hexagonal crystal system). S12 mainly contained Co (a cubic crystal system), Cr21W2C6 (a cubic crystal system), ZrO_2_ (a monoclinic crystal system), and Co (a hexagonal crystal system).

#### 3.1.1. S1 Welding Wire

**Figure 2:**
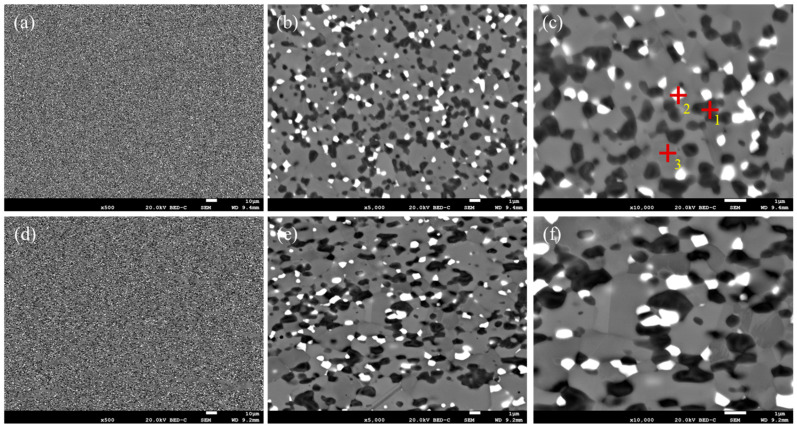
Microstructure of S1 alloy: (**a**–**c**) low–high-magnification morphology in transverse direction, where +1, +2, and +3 were the points for spectral analysis; (**d**–**f**) low–high-magnification morphology in longitudinal direction.

**Figure 3:**
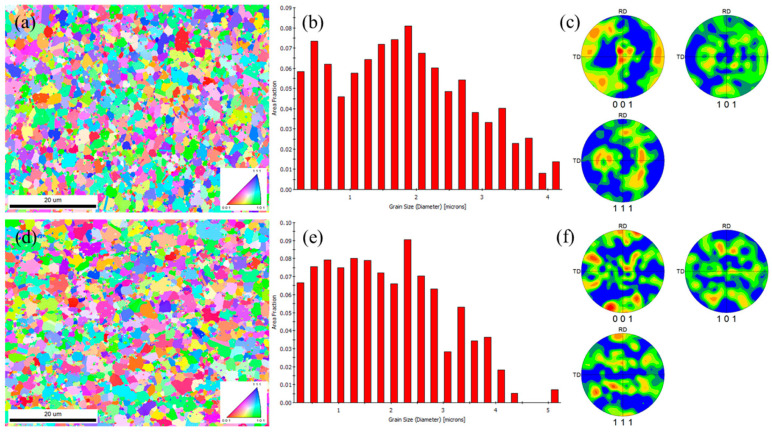
Microstructure, grain distribution, and pole figure of S1 alloy (**a**–**c**) in the transverse direction and (**d**–**f**) longitudinal direction.

The S1 alloy was observed via both transverse and longitudinal sampling. The microstructure and composition were consistent, indicating that this alloy had isotropy, as shown in Figure 2. The matrix of the welding wire was composed of CoCr, and the main phases, such as CrCo and CoCrW, were uniformly distributed in the matrix (Table 2). The grain size in both directions was within 3 μm, and the distribution was relatively uniform (Figure 3). The XRD pattern of the S1 alloy layer was combined. As shown in Figure 1, the main phases of the alloy overlay were CoCr, γ-Co, and (Cr, Fe)7C3. Among them, CoCr had a hexahedral structure, γ-Co had a face-centered cubic (FCC) structure, and (Cr, Fe)7C3 was a hard strengthening phase with a high melting point, high hardness, and corrosion resistance. Based on SEM image statistics using Image J 1.8.0, the proportions of (Cr, Fe)7C3 in the transverse and longitudinal directions of the S1 alloy layer were 6.74% and 7.07%, respectively.

#### 3.1.2. S6 and S12 Welding Wires

**Figure 4:**
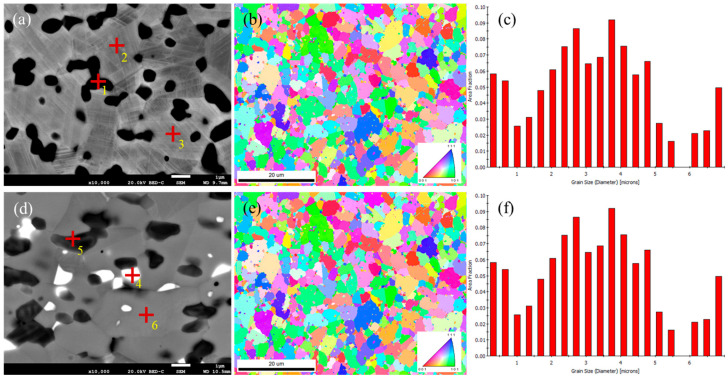
(**a**–**c**) Microstructure and grain distribution of S6 alloy in transverse direction; (**d**–**f**) microstructure and grain distribution of S12 alloy in transverse direction, where +1, +2, +3, +4, +5, and +6 were the points for spectral analysis.

Figure 4 shows the eutectic carbides and block carbides. In the backscattered images, the carbides were divided into two types: dark gray and bright white, which were more pronounced in the backscattered images of the block carbides. In the cobalt-based high-temperature alloys, the carbides mainly included M23C6, M7C3, M6C, and MC, so different colors may represent different carbides. Table 3 shows the compositions of the S6 and S12 alloys. EDS element scanning is not accurate in detecting carbon content, but it is more accurate in detecting metal element content. The EDS elemental point scan results showed that the main elements in the dendrites were Co and Cr. The main elements in the dark gray of S6 were Co, Cr, and W. The main elements in the black carbides were also Co, Cr, and W, but their W content was much higher than that in the dark gray carbides. The main elements in the S12 dark gray carbides were Co, Cr, and W, with a significant increase compared with the matrix Cr element. The main elements in the bright white carbides were also Co, Cr, and W, but their W content was much higher than that in the dark gray carbides. This element distribution also verifies the crystallization process, first forming a cobalt-based solid solution rich in cobalt and then forming a eutectic structure of cobalt and carbides. M23C6 and M7C3 are Cr-rich carbides, while M6C is a W-rich carbide. The W content in S12 had significantly increased compared with S6. Solid-solution strengthening and aging precipitation strengthening were the main strengthening mechanisms of the high-temperature alloys. Elements such as Cr and W can play a role in solid-solution strengthening when dissolved in a cobalt matrix. M23C6, M7C3, and M6C carbides can play a role in aging precipitation strengthening. These carbides have high hardness and can improve the hardness and wear resistance. In addition, the grain sizes of S6 and S12 were comparable, with an average of 3 μm, as shown in Figure 4c,f.

Co-based alloys typically contain a mass fraction of 0.25–3% C [33]. When the C content is less than 2 %, the alloy is hypoeutectic, and when the carbon content is greater than 2 %, the alloy is hypereutectic [34]. The C content in the S12 alloy was 1.4%, making it a hypoeutectic alloy. During the solidification process, the Co-based solid solution first precipitated from the liquid phase. As the Co content in the liquid phase decreased, the remaining liquid phase met the conditions for eutectic composition, resulting in the formation of a eutectic structure of cobalt and carbides through a eutectic reaction [35].

### 3.2. Influence of Welding Wire on Microstructure of the Deposited Layer

#### 3.2.1. Microstructure of S1 Welding Wire

**Figure 5:**
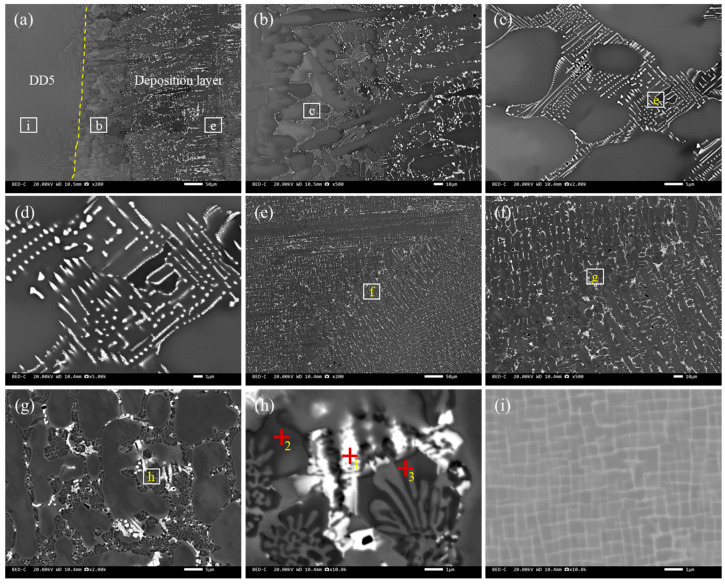
The backscatter microstructure of the deposited S1 layer: (**a**) macroscopic photos of the weld layer, where the dashed line is the boundary between the substrate and the deposited alloy layer; (**b**) the interfacial microstructure; (**c**,**d**) the welding microstructure and its high-magnification microstructure near the interface; (**d**–**g**) the microstructure at the center of the weld layer; (**h**) the eutectic structure in the overlay layer, where +1, +2, and +3 were the points for spectral analysis; and (**i**) the base material, where (**e**–**h**) represent enlarged images of each region.

**Figure 6:**
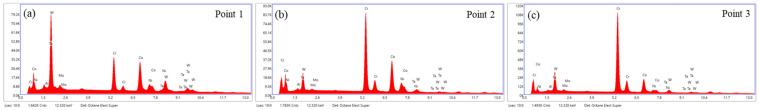
EDS point scanning of deposited S1 layer.

Figure 5 shows the microstructure of the deposited S1 layer. The dendrites in the S1 layer were relatively long and showed a trend of multi-directional growth. The distance between the dendrites was wide, and the gray phase surrounded by the dendrites manifested as long, rod-like and large, irregular agglomerates. The white phase in Figure 5b represents the dendritic crystals precipitated during the cooling process, while the gray phase represents the eutectic structure precipitated between the dendrites when the alloy layer cooled to the eutectic transformation temperature. Figure 6 shows the point scan results of the light gray, dark gray, and white microstructure of the S1 alloy layer. The light gray structure was a solid solution phase containing alloy elements such as Cr, Fe, W, Mn, etc., with CoCr as the matrix. The dark gray structure was a face-centered cubic solid solution with γ-Co as the matrix. The white tissue was mainly composed of eutectic carbides of (Cr, Fe)7C3. The magnified images revealed numerous black, fine, line-like structures distributed along the edges of the dark gray and white structures in the S1 alloy layer. The presence of black, fine, line-like structures made the contour of the S1 alloy layer structure clearer and more distinct. The eutectic structure of (Cr, Fe)7C3 exhibited different morphologies, which were caused by the contents of elements such as Cr, Fe, and C in the eutectic carbide structure [36].

#### 3.2.2. S6 Welding Wire

**Figure 7:**
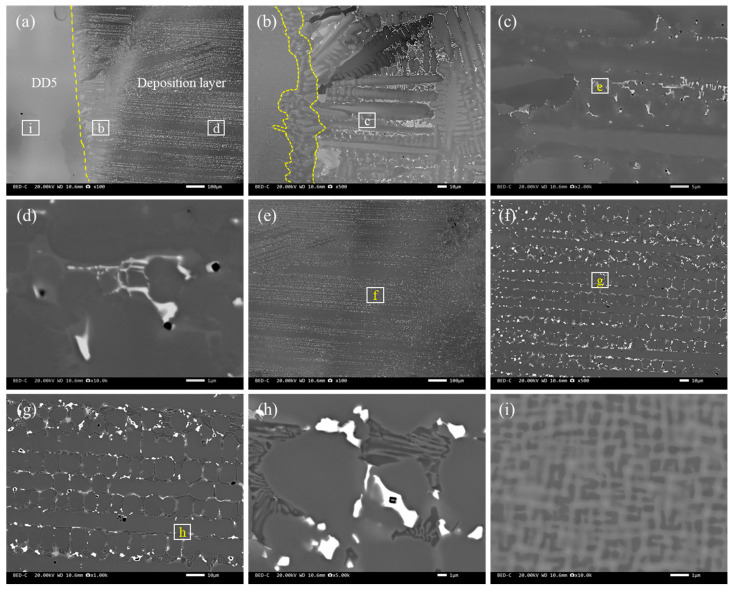
The backscatter microstructure of the deposited S6 layer: (**a**) macroscopic photos of the weld layer, where the dashed line is the boundary between the substrate and the deposited alloy layer; (**b**) the interfacial microstructure; (**c**,**d**) the welding microstructure and its high-magnification microstructure near the interface; (**d**–**g**) the microstructure at the center of the weld layer; (**h**) the eutectic structure in the overlay layer; and (**i**) the base material, where (**e**–**h**) represent enlarged images of each region.

Figure 7 shows the microstructure of the deposited S6 layer. After welding, the welding interface could be clearly observed. The S6 welding layer, composed of precipitated phases between dendrites, was on the right side. The matrix was on the left side, and the grain size of the matrix near the interface was significantly reduced (Figure 7a). This was the normalized fine-grain zone generated near the substrate during the arc-welding process. During the arc-welding process, no coarse-grain structure was generated near the welding interface, which greatly benefited the sample’s performance. Figure 7b shows the low-magnification microstructure at the interface between S6 and the substrate, and an intermediate layer was observed at the interface.

Figure 7c shows the microstructure near the interface between S6 and the substrate at a low magnification, and a layer of planar crystals near the interface was formed. This is because the temperature of the substrate was relatively low during the first layer of arc welding, resulting in a significant degree of undercooling. At this point, the undercooling curve did not intersect with the solidification curve, and the solidification rate at each position was the same, resulting in a planar crystal state. Then, the undercooling decreased and intersected with the solidification curve, gradually producing cellular and equiaxed crystals. Figure 7d–f show the microstructures at different magnifications at the interface. An intermediate layer was formed at the weld interface in the original sample, and the distribution of this layer was not very uniform. The thickness of the intermediate layer was relatively large at some grain boundaries inside the plane crystal, and there were only a few carbides at the grain boundaries, which were connected by a network of carbides at the upper end. This indicated that the growth of the intermediate layer was related to eutectic carbides, and this layer “absorbed” elements from the network carbides via grain boundaries, thereby undergoing growth. Therefore, the growth of the intermediate layer was related to thermal diffusion, which was particularly evident in the aging treatment.

#### 3.2.3. S12 Welding Wire

**Figure 8:**
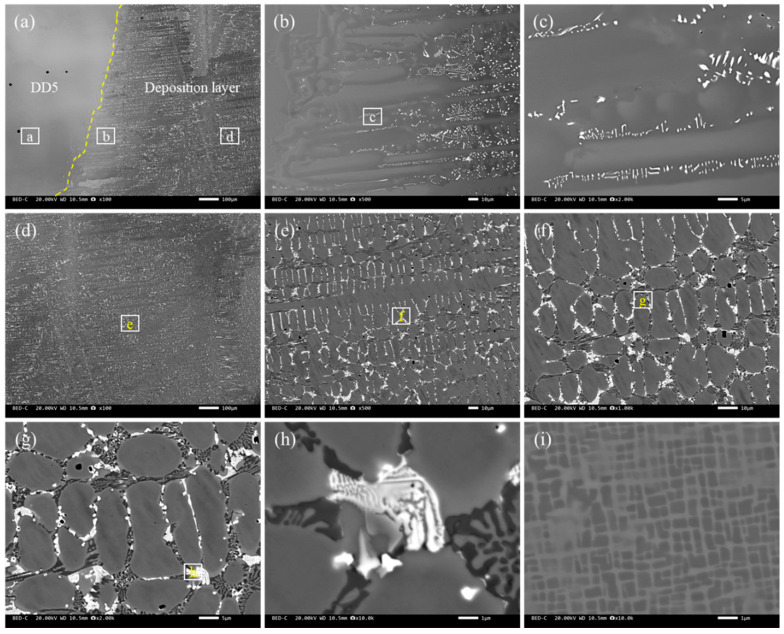
The backscatter microstructure of the deposited S12 layer: (**a**) macroscopic photos of the weld layer, where the dashed line is the boundary between the substrate and the deposited alloy layer; (**b**) the interfacial microstructure; (**c**,**d**) the welding microstructure and its high-magnification microstructure near the interface; (**d**–**g**) the microstructure at the center of the weld layer; (**h**) the eutectic structure in the overlay layer; and (**i**) the base material, where (**e**–**h**) represent enlarged images of each region.

Figure 8 shows the microstructure of the deposited S12 layer. The alloy layer formed a good metallurgical bond with the matrix, and no metallurgical defects, such as cracks, pores, or lack of fusion, were observed. The microstructure of the alloy layer was dense, consisting of planar crystals, coarse columnar dendrites, and fine, dense equiaxed dendrites from the bottom to the middle. During the melting process, the temperature at the bottom of the molten pool was high and heat dissipation was slow, resulting in a large positive temperature gradient. As the molten metal cooled, a certain thickness of planar crystals was first formed. The alloy composition varied during solidification due to the loss of latent heat of crystallization. Some high-melting-point solute elements solidified first, while low-melting-point solute elements accumulated at the interface front, leading to the preferential growth of some grains and the inhibition of other grains, forming columnar dendrites with obvious directional characteristics [37]. The temperature gradient decreased as the latent heat of crystallization continued to dissipate, and significant component undercooling occurred at the interface front, leading to the formation of equiaxed dendrites. Each dendrite had almost the same potential energy to drive growth, thereby hindering the growth of columnar dendrites and forming dense equiaxed dendrites. In addition, the laser cladding alloy layer had narrower planar crystal bands and denser, finer dendritic structures compared with the microstructure of the alloy layer. This difference was mainly attributed to the higher heat input of tungsten gas-shielded arc welding, the greater fluctuation in the liquid composition of the alloy layer, the slower cooling rate, and the easier formation of coarse dendritic structures [38].

**Figure 9:**
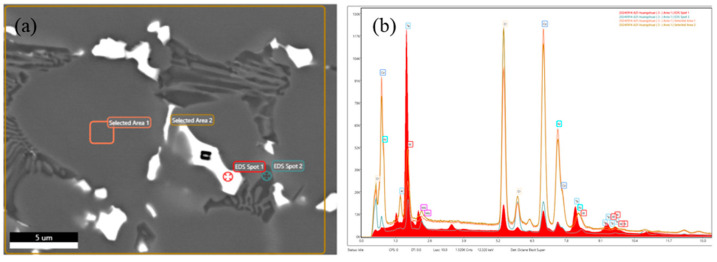
Microstructure and composition of S12 alloy layer: (**a**) microscopic photos of weld layer and (**b**) components.

Figure 9 shows the composition analysis of the central dendrites in the S12 layer. The dendrites were mainly composed of Cr, Co, and W, with an uneven distribution. Compared with the intragranular segregation, there was a segregation phenomenon of a large amount of Cr and a small amount of W at the grain boundary, which may be due to the strong affinity between Cr, W, and C, forming stable carbides and precipitating along the grain boundary. As an element that accelerates the formation rate of austenite, Co had significantly fewer grain boundary sites than intragranular sites. This was because the grain boundary sites had higher energy and, when they interacted with Cr and W, they reduced the solid solubility of Cr and W in the matrix; promoted the formation of Co, Cr, and W compounds; and reacted with C to form various carbide precipitates [39]. In addition, tungsten gas-shielded arc welding had a higher heat input and fusion ratio compared with laser cladding, which led to more segregation of the deposited metal due to the dilution effect of the base material, with more segregation of Cr and W.

**Figure 10:**
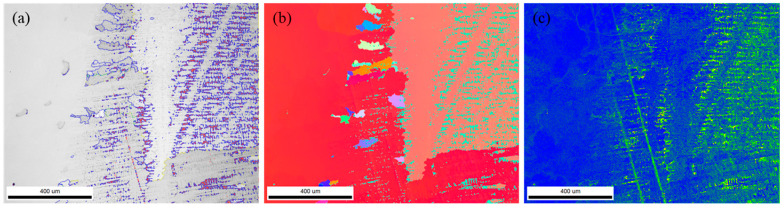
Microstructure S12 alloy layer: (**a**) IQ image, (**b**) IPF image, and (**c**) KAM image.

As the angle between the preferred growth direction of dendrites and the temperature gradient direction increased, the incubation time for the instability of the grain interface increased, and the stability of the interface was enhanced, as shown in Figure 10. For the morphological evolution of dendrites with different crystal orientations, the larger the angle between the preferred growth direction of dendrites and the temperature gradient direction, the more severe the asymmetric growth of secondary arms of dendrites [40].

### 3.3. Microhardness Distribution of Different Welding Wire Layers

**Figure 11:**
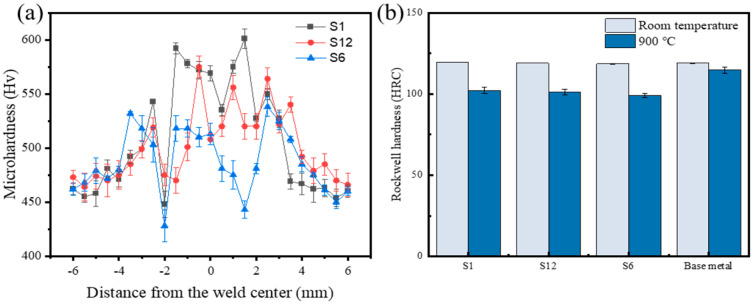
The microhardness of the different deposited layers: (**a**) the room-temperature microhardness distribution; (**b**) the microhardness in the center of the deposited layer at high temperatures.

Figure 11a shows the microhardness distribution curves of different deposited alloy layers at room temperature. The microhardness of the alloy layer increased with the distance from the interface, showing a distribution pattern of the highest in the center of the deposition layer, followed by the interface, and the lowest at the base material. The average microhardness values of the S1, S6, and S12 deposited alloy layers were 570 HRC, 510 HRC, and 530 HRC, respectively, with a significant fluctuation trend in the deposited layer area. The analysis of the microstructure and phase structure of the deposited layer revealed that the rapid cooling of the molten pool after welding resulted in fine-grain strengthening, the solid-solution strengthening effect caused by the dissolution of Mn, Cr, W, Ni, and other elements in the alloy into dendrites to form supersaturated solid solutions, and the hard strengthening effect caused by the dispersion of (Cr, Fe)7C3 eutectic compounds all contributed to the high hardness of the alloy layer surface [41].

The deposited layer of GTAW was greatly affected by thermal cycling, with a high heat input, a coarser dendritic structure, and an intensified dilution effect of the alloy layer resulting in low microhardness and significant fluctuations in the alloy layer. Meanwhile, the uneven heat distribution led to an uneven microstructure and significant fluctuations in the alloy layer’s microhardness due to the non-equilibrium solidification process of the alloy. The microhardness on the side closer to the interface was slightly increased compared with the matrix, which may have resulted from the influence of thermal cycling and the dilution effect of alloy elements on both sides of the interface.

Figure 10b shows the microhardness distribution curves of different deposited alloy layers at 900 °C. The average hardness values of the S1, S6, and S12 alloy overlays were 103.2 HRC, 99.1 HRC, and 101.7 HRC, respectively. Compared with the room-temperature microhardness, the deposited layer became softer and the hardness decreased in the high-temperature environment. The decreases in hardness were basically the same for the three types of welding wires.

### 3.4. Friction and Wear Performance of Different Deposited Layers

**Figure 12:**
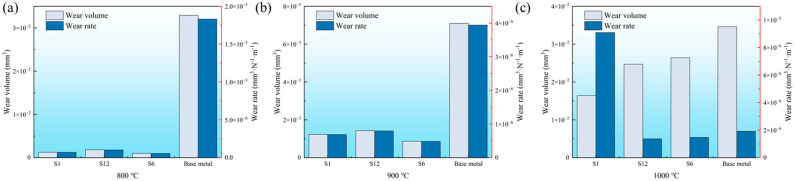
Wear resistance of three different deposited layers at different temperatures: (**a**) 800 °C; (**b**) 900 °C; (**c**) 1100 °C.

Figure 12 shows a comparison of the wear performance of alloy layers. In a high-temperature test at 800 °C, the wear volume of the S1 alloy layer was 1.26 × 10^−3^ mm^3^, with a wear rate of 6.99 × 10^−7^ mm^3^ N^−1^·m^−1^. The wear volume of the S6 alloy layer was 1.83 × 10^−3^ mm^3^, and the wear rate was 10.2 × 10^−7^ mm^3^ N^−1^·m^−1^. The wear volume of the Stellite 12 alloy layer was 1.01 × 10^−3^ mm^3^, with a wear rate of 5.62 × 10^−7^ mm^3^ N^−1^·m^−1^. The wear resistance of the three deposited layers was significantly improved compared with the base material (wear volume: 3.29 × 10^−2^ mm^3^; wear rate: 1.83 × 10^−5^ mm^3^ N^−1^·m^−1^), with S1 and S6 showing better performance.

In a high-temperature test at 900 °C, the wear volume of the S1 alloy layer was 1.23 × 10^−3^ mm^3^, with a wear rate of 6.86 × 10^−7^ mm^3^ N^−1^·m^−1^. The wear volume of the S6 alloy layer was 1.43 × 10^−3^ mm^3^, and the wear rate was 7.94 × 10^−7^ mm^3^ N^−1^·m^−1^. The wear volume of the S12 alloy layer was 8.68 × 10^−4^ mm^3^, and the wear rate was 4.82 × 10^−7^ mm^3^ N^−1^·m^−1^. The wear resistance of the S6 deposited layer had the best performance.

In a high-temperature test at 1000 °C, the wear volume of the S1 alloy layer was 16.4 × 10^−3^ mm^3^, with a wear rate of 9.09 × 10^−6^ mm^3^ N^−1^·m^−1^. It presented a sharp drop in wear resistance because phase instability and carbon burnout occurred [42,43]. The wear volume of the S6 alloy layer was 2.47 × 10^−3^ mm^3^, and the wear rate was 1.37 × 10^−6^ mm^3^ N^−1^·m^−1^. The wear volume of the S12 alloy layer was 2.64 × 10^−3^ mm^3^, and the wear rate was 1.47 × 10^−6^ mm^3^ N^−1^·m^−1^. The wear resistance of the deposited layer significantly decreased with the increase in the service temperature. S1 had a similar wear rate to that of the base material, and S6 had the lowest wear rate and the best wear resistance.

According to the microhardness analysis, the S6 deposited alloy layer had the best microhardness, and the wear resistance was proportional to its hardness. The microstructural analysis revealed that the deposited layer had the same phase structure. However, the welded alloy layer had a denser and finer dendritic structure, and the distribution of dendritic components was more uniform, which also increased the wear resistance.

### 3.5. Tensile Properties of Three Different Welding Wire Overlay Joints

**Figure 13:**
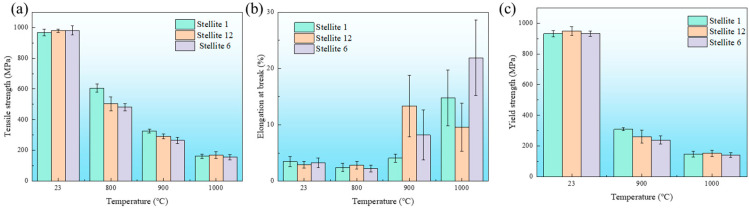
Tensile properties of three types of welding wire overlay joints at different temperatures: (**a**) tensile strength, (**b**) elongation rate, and (**c**) yield strength.

Figure 13 shows the tensile strengths of the three types of welding wire joints. At room temperature, the tensile strengths of the three types of welding wires were comparable, ranging from 975 to 980 MPa. When the temperature rose to 800 °C, the strengths of the S1, S6, and S12 alloy joints were 605 MPa, 482 MPa, and 503 MPa, respectively. When the temperature rose to 900 °C, the strengths of the S1, S6, and S12 alloy joints were 325 MPa, 264 MPa, and 292 MPa, respectively. When the temperature rose to 1000 °C, the strengths of the S1, S6, and S12 alloy joints were 161 MPa, 155 MPa, and 168 MPa, respectively. As the testing temperature increased, the strength of the joint obviously decreased. The trends of changes in elongation and tensile strength were opposite. Moreover, the fluctuation in elongation at high temperatures was greater. The trend of change in the yield strength was consistent with that of the change in tensile strength, and both strengths decreased with increasing temperature.

## 4. Conclusions

(1) The main phases of the three deposited alloy welding wires were CoCr, γ-Co, and (Cr, Fe)7C3. Among them, CoCr was a hexahedral structure, γ-Co was a face-centered cubic structure, and (Cr, Fe)7C3 was a hard strengthening phase. The average grain size was 3 μm.

(2) Three types of deposited alloy welding wires formed a good metallurgical bond with the substrate without any metallurgical defects. The microstructure of the deposited layer was dense, consisting of planar crystals, coarse columnar dendrites, and fine, dense equiaxed dendrites from the bottom to the middle. A large amount of eutectic structure was distributed throughout the deposited layer.

(3) The hardness of the three deposited alloy layers increased with increasing distance from the interface, showing a distribution pattern of highest in the center of the deposited layer, followed by the interface, and lowest at the base metal. The average hardness values of the S1, S6, and S12 alloy layers were 103.2 HRC, 99.1 HRC, and 101.7 HRC, respectively, with a significant fluctuation trend observed in the deposited layer.

(4) The wear resistance of the three types of deposited alloy was significantly improved. The wear resistance significantly decreased with an increase in the service temperature. At room temperature, the tensile strength of the three deposited welding wires was similar. The strength of the joint gradually decreased as the testing temperature increased.

## Figures and Tables

**Table 1:** Composition and melting points of different welding wires.

	Co	W	C	Cr	Ni	Fe	Mn	Mo	Si	Melting Point
S1	Bal.	11.78	1.97	28.26	1.94	0.56	0.16	0.22	0.64	1248~1290 °C
S6	Bal.	4.86	1.14	28.78	2	1.97	0.36	0.45	1.28	1250~1360 °C
S12	Bal.	8.67	1.5	29.4	2.62	2.61	0.27	0.55	1.2	1225~1280 °C

**Table 2:** Composition of S1 alloy (at. %).

Element	Point 1	Point 2	Point 3
Cr	67	26.91	23.2
Fe	0.45	0.58	0.69
Co	29.33	53.13	69.83
Ni	0.75	1.63	2.39
Mo	0.1	0.72	0.14
W	2.38	17.03	3.75

**Table 3:** Compositions of S6 and S12 alloys.

	Point 1	Point 2	Point 3	Point 4	Point 5	Point 6
Cr	76.7	28	28.98	30.43	70.23	26.62
Fe	0.92	2.25	2.18	1.82	1.55	3.15
Co	20.41	64.99	63.48	47.56	24.79	63.56
Ni	0.36	2.09	2.12	2.44	0.95	3.02
Mo	0.3	0.3	0.7	1.72	0.33	0.28
W	1.3	2.37	2.53	16.02	2.15	3.38

## Data Availability

The original contributions presented in this study are included in the article. Further inquiries can be directed to the corresponding authors.
